# An Unusual Cause of Chylothorax after Esophagectomy

**DOI:** 10.1055/s-0040-1713417

**Published:** 2020-09-10

**Authors:** John Mathew Manipadam, Chokkappu S. Kumar, Rajesh Antony, Abhishek Yadav, H. Ramesh

**Affiliations:** 1 Department of GI surgery and liver transplantation, VPS Lakeshore Hospital and Research Centre, Kochi, Kerala, India; 2 Department of Radiology, VPS Lakeshore Hospital and Research Centre, Kochi, Kerala, India; 3 Department of Liver Transplantation, VPS Lakeshore Hospital and Research Centre, Kochi, Kerala, India

**Keywords:** chylothorax, esophagectomy, chyle

## Abstract

Chylothorax due to inadvertent thoracic duct injury after esophagectomy is a well-known complication and requires careful postoperative management and timely intervention to prevent potential morbidity and mortality. We present a case of high-output chylothorax after esophagectomy where the source of chyle leak was not in the thorax.

Thoracic duct injury with ensuing chylous leak following esophagectomy for cancer has an incidence ranging from 0.4 to 9% and is a potential cause for morbidity and mortality if not rectified 1 2

Chyle is rich in fluid, proteins, lipids, and lymphocytes and patients who have a significant leak get depleted nutritionally and immunologically. We present an unusual case of chylothorax after esophagectomy and how we rectified it.

## Case Presentation

A 39-year-old male patient was evaluated at another center for dysphagia to solids and weight loss for 6 months. Upper gastrointestinal (GI) endoscopy showed an ulceroproliferative growth starting at 33 cm and extending up to 42 cm involving the gastroesophageal junction. Biopsy was positive for adenocarcinoma. Contrast-enhanced computed tomography (CECT) abdomen done at the same center was suggestive of asymmetrical mural wall thickening involving the lower one-third of the esophagus, gastroesophageal junction. and extending onto the proximal stomach . He was initiated on neoadjuvant chemotherapy with docetaxel, cisplatin, and 5 fluorouracil and received three of those cycles. He subsequently came to us for further management after having done a repeat imaging which showed marked response to treatment with a diffuse thickening of the distal esophagus remaining. General and systemic examination revealed no abnormalities except for a low body mass index (BMI). Repeat upper GI endoscopy showed the growth starting at 33 cm and extending along the lesser curvature up to 2 cm below the gastroesophageal junction.

In view of the marked response to chemotherapy demonstrated on two successive imaging, we planned for a radical thoracoscopic esophagectomy. Cardiopulmonary and anesthetic fitness was ascertained. We initiated Incentive spirometer for respiratory fitness and high-protein supplements in view of low BMI. Patient was counselled, informed consent was obtained, and scheduled for a thoracoscopic esophagectomy followed by gastric/colonic pull up.

Initial staging laparoscopy in supine position did not reveal any distant metastases. Subsequently, we thoracoscopically completed esophagectomy in the prone position according to our standard technique. 3 As expected from the imaging, the ulceroproliferative growth was involving the lower esophagus, gastroesophageal junction, and initial 2 cm of cardia and lesser curvature. The tumor was not adherent to the surrounding pericardium, aorta, or diaphragm and could be removed with a safe margin along with an adequate extended two-field lymphadenectomy. The thoracic duct was excised in its course along the tumor in the thorax and the proximal and distal ends were doubly clipped securely in the thorax in order to prevent a chyle leak. We at our center, preferably ligate and excise the thoracic duct during esophagectomy whenever possible (unless it is technically not feasible and patient is unstable). The reason for this strategy is supported by evidence and is two fold: (1) it significantly reduces the incidence of postoperative chylothorax, 4 (2) it significantly increases the lymph node yield, especially around the region of the thoracic duct. 5

A standard gastric pullup and cervical esophagogastric anastomosis was subsequently performed.

Postoperatively patient had a high output from the right intercostal drainage tube from postoperative day 2 which turned chylous once jejunostomy feeds were started. Drain fluid triglyceride levels were high confirming a chylous leak. We initiated conservative management with total parenteral nutrition, nil by mouth, and octreotide injections thrice a day. However, the right intercostal drainage output was persistently high in the range of 1.5 to 2 L/day. We refrained from reexploring immediately in view of the fact that we had already identified and clipped the thoracic duct intraoperatively.

**Fig. 1:**
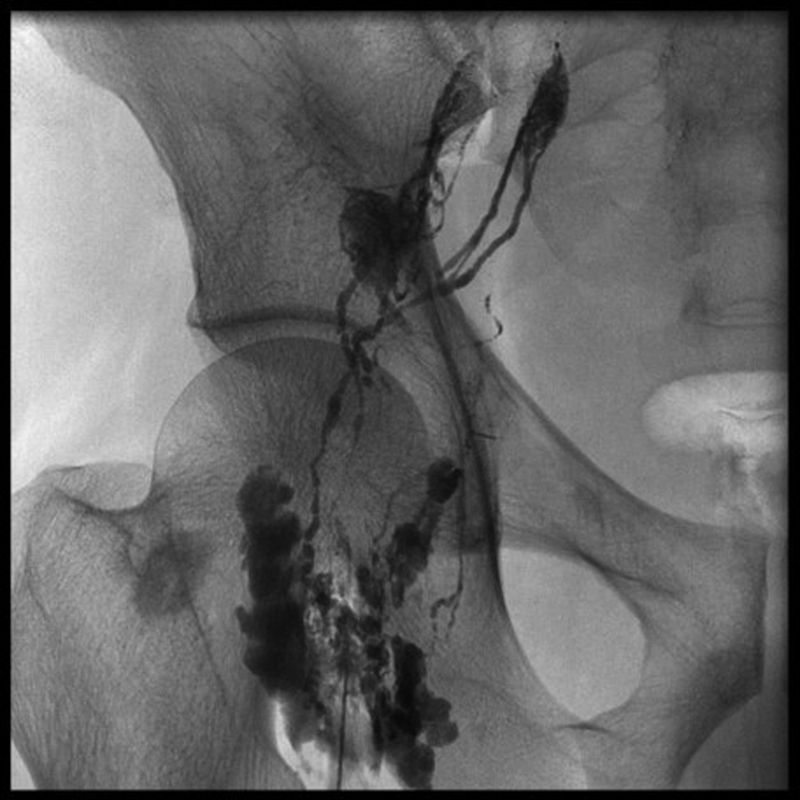
Groin lymphangiogram with contrast plus lipiodol.

**Fig. 2:**
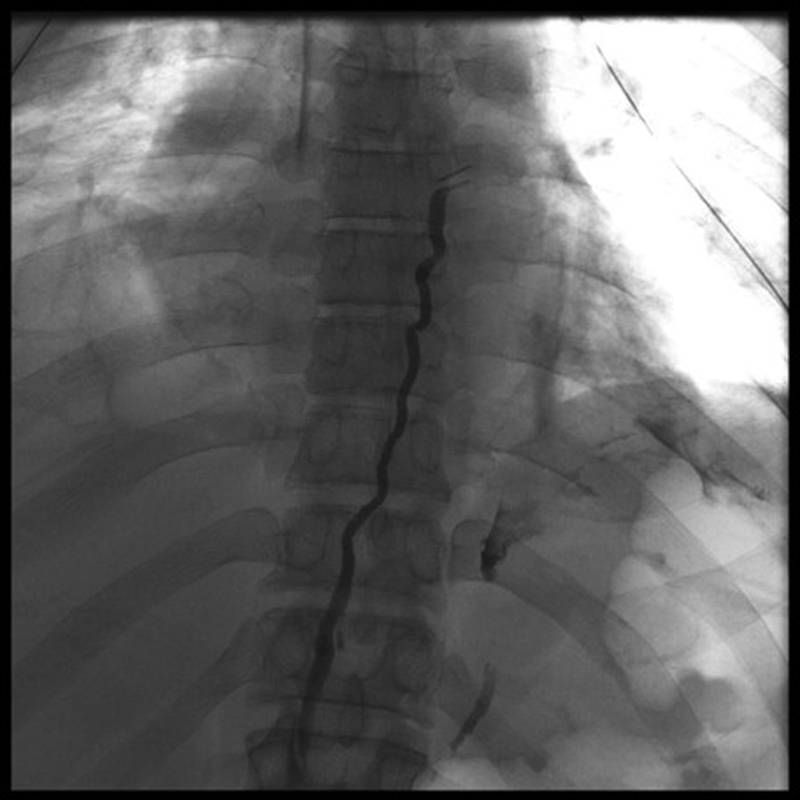
Thoracic duct embolization with lipiodol showing clipped thoracic duct and site of leak in the left subdiaphragmatic region.

On postoperative day 9, we attempted radiological identification and embolization of the thoracic duct leak by injecting lipiodol in and around the bilateral superficial inguinal lymph nodes using ultrasound guidance ( Fig. 1 ) and subsequent c-arm visualization of the site of leak from the thoracic duct. 6 To our surprise, we found that the thoracic duct was opacifying well up to the site of the clip in the thorax, but without any leak from it. The only leak that could be demonstrated was a minor one in the left subdiapghramatic location from an adjacent lymphatic tributary ( Fig. 2 ). A subsequent computed tomography (CT) thorax and abdomen on the same day confirmed the same findings. As there was no significant free fluid in the abdomen on imaging and the right subhepatic abdominal drain was nil, we decided on further conservative management by adding intravenous octride infusion and oral midodrine as some evidence has suggested for refractory chylothorax. However, there was no respite from the high output chylothorax (more than 1 L) though initially it came down to 500 to 750 mL per day ( Table 1 ).

**Table 1:** Trend of intercostal drain output

Postoperative day	Right chest drain	Left chest drain	Management
1	250	100	
2	650	600	
3	1,200	900	
4	2,150	550	Peptamen feeds, µt oil, octreotide sc 200 µg tid
5	2,750	250	Oral dye study normal, Ryles tube removed
6	2,050	10	Started TPN, allowed oral sips, octreotide continued
7	1,700	50	
8	1,600	50	
9	1,450	50	Lymphangiogram and lipiodol injection into cisterna chyli
10	490	30	
11	790	Nil	Left chest drain removed
12	700		Stopped TPN, oral liquids 100 mL per hour
13	850		
14	1,030		Injection octreotide IV 100 µg tid, tab midodrine 5 mg bd
15	1,000		Semisolid diet, midodrine 7.5 mg tid
16	980		Injection octreotide 250 µg IV tid., midodrine 10 mg tid
17	500		Fat-free diet
18	1,560		Fat-free diet

Abbreviations: bd, twice a day; IV, intravenous; sc, subcutaneous; tid, thrice a day; tpn, total parenteral nutrition.

On postoperative day 18, after several sessions of thorough preoperative counselling with the patient and relatives, we decided to reexplore the patient. Anticipating dense adhesions during the unfriendly third postoperative week, we decided to explore the abdomen rather than the thorax first. The transverse colon had densely sealed off the supracolic compartment form the rest of the abdomen. As soon as we released it, there was a gush of chyle of about 2 L, and we could slowly identify after a lot of suction and irrigation that the source of leak was from robust lymphatic vessels in the retroperitoneum in the left subdiaphgrmatic area where the splenic artery lymph nodes were dissected out as a part of the standard d2 lymphadenectomy.

Once these vessels were secured with repeated ligatures of 2–0 prolene, there was no more active chyle leak in sight. There was no active chyle draining from the thorax into the abdomen through the hiatus or into the right chest tube.

Patient was extubated after the operation and the right ICD output reduced significantly in the next few days; so it was subsequently removed 6 days after the reexploration. Patient was discharged on a normal diet and is doing well. Histopathology was reported as t3n1 adenocarcinoma with partial response to chemotherapy.

## Discussion

We report this case for its unusual presentation of chyle leak through the thorax though the actual site was in the abdomen from a retroperitoneal lymphatic and not from the thoracic duct. This was also the reason for the delay in reexploring the patient, since we were confident about identifying and clipping the thoracic duct intraoperatively. Hence we decided initially to manage conservatively and then to radiologically embolize any minor leak from tributaries with lipiodol.

The radiological investigation also pointed out to the intactness of the thoracic duct and showed the site of the leak in the left subdiaphragmatic area. But since the abdominal drain was nil and there was no significant free fluid on imaging, we were hesitant and tried conservative management again for a few days. The reason for this was obviously the transverse colon which had sealed off the site of leak from the rest of the abdomen and the chyle found its way into the thorax through the widened hiatus.

## Conclusion

What we conclude from this case is that in rare instances of chylothorax after esophagectomy where one has already ligated the thoracic duct intraoperatively, it may be wise to keep a low threshold for reexploring from the abdominal side first. We believe that one can tackle the site of leak near thoracic duct origin in the abdomen where there is a more constant anatomy in these cases where there is a leak from a variant anatomy.
